# Life on the Rail

**Published:** 1861-11

**Authors:** 


**LIFE ON THE RAIL.**—The voluble Yankee would rather use a dozen words than one; the dignified, pompous and taciturn Englishman employs the smallest number of syllables possible when he wishes to express himself; still, he takes good care that the words he does use shall tell his whole story unmistakably and full. To him "bus" tells as much as an omnibus, a saving of sixty-six and two thirds per cent. He travels by "rail," never by railroad. But, perhaps, if John Bull talked more, he would be kept wider awake, and thus be a gainer in the long run. For example, a Liverpool paper reported recently that fifty-one lives were lost by a railway accident near London, and a later packet brings the news that "another" accident occurred on a suburban train, by which thirteen more lives were lost, and fifty-one persons wounded. These calamities were traced to a drowsy flagman and a sleepy switch-tender. Our transatlantic cousins had better deputize half a dozen railway directors to visit our country and inquire into the management of the New-Jersey Railroad Co., under the Presidency of J. S. Darcy, Esq., and J. P. Jackson. They would learn the extraordinary fact, that since its organization, thirty-six millions of persons have ridden in their cars without the loss of life or limb, while occupying their proper seats. Such fidelity to duty on the part of the managers and employés of the road certainly merits public appreciation and patronage. The above results were obtained chiefly by two plans of conduct: first, the windows are so constructed that a passenger can not put out his arm or his head without maintaining a most uncomfortable position of body. Second, a liberal "bonus" is paid every three months to every employé on whose "route" no accident has happened, with a fine or dismission if any thing goes wrong for want of diligence. Let every railway president and director make a note of this; and emulate the carefulness of this, one of the very oldest railroad companies in the nation. And it may be worth the life of the reader to remember, while he keeps his seat in the cars, he has ninety-nine chances in a hundred of escaping injury altogether.

